# Do Herbivores Eavesdrop on Ant Chemical Communication to Avoid Predation?

**DOI:** 10.1371/journal.pone.0028703

**Published:** 2012-01-03

**Authors:** David J. Gonthier

**Affiliations:** Department of Environmental Sciences, University of Toledo, Toledo, Ohio, United States of America; University of Tartu, Estonia

## Abstract

Strong effects of predator chemical cues on prey are common in aquatic and marine ecosystems, but are thought to be rare in terrestrial systems and specifically for arthropods. For ants, herbivores are hypothesized to eavesdrop on ant chemical communication and thereby avoid predation or confrontation. Here I tested the effect of ant chemical cues on herbivore choice and herbivory. Using *Margaridisa* sp. flea beetles and leaves from the host tree (*Conostegia xalapensis*), I performed paired-leaf choice feeding experiments. Coating leaves with crushed ant liquids (*Azteca instabilis*), exposing leaves to ant patrolling prior to choice tests (*A. instabilis* and *Camponotus textor*) and comparing leaves from trees with and without *A. instabilis* nests resulted in more herbivores and herbivory on control (no ant-treatment) relative to ant-treatment leaves. In contrast to *A. instabilis* and *C. textor*, leaves previously patrolled by *Solenopsis geminata* had no difference in beetle number and damage compared to control leaves. Altering the time *A. instabilis* patrolled treatment leaves prior to choice tests (0-, 5-, 30-, 90-, 180-min.) revealed treatment effects were only statistically significant after 90- and 180-min. of prior leaf exposure. This study suggests, for two ecologically important and taxonomically diverse genera (*Azteca* and *Camponotus*), ant chemical cues have important effects on herbivores and that these effects may be widespread across the ant family. It suggests that the effect of chemical cues on herbivores may only appear after substantial previous ant activity has occurred on plant tissues. Furthermore, it supports the hypothesis that herbivores use ant chemical communication to avoid predation or confrontation with ants.

## Introduction

The role of predators limiting herbivores and indirectly benefiting plants has formed an important foundation of ecology [1]–[2]. Today, researchers are now interested in understanding how predators indirectly benefit plants, whether it is through density-mediated (lethal effects) or trait-mediated (non-lethal) effects on prey (herbivores) [3]. For ants (Hymenoptera: Formicidae) on plants, an overwhelming majority of ant-exclusion studies, as well as, recent meta-analyses provide empirical evidence that ants deter herbivores from damaging plants and increase plant fitness [2], [4]–[7] but see exceptions [8]. The importance of ants to plants is also exemplified in that one-third of tropical woody plant species have extra-floral nectaries that attract ants or specialized domatia that house ants on their tissues [9]. And there is a fascinating diversity of arthropods that gain protection from ants through mimicry or offering rewards for ant services [10]. Yet few studies focus on how ants deter herbivores and it is often assumed that direct density-mediated interactions or aggressive removal of herbivores is enough to explain the effectiveness of ant defenses. In contrast, trait-mediated effects may also contribute to the success of ant defenses [11]–[14], but have received much less attention.

Many predators produce trait-mediated effects on prey that have equally strong or even stronger effects than direct density-mediated interactions [3]. The intensity of predator cues given off can induce a threat-sensitive response (graded linear response to cues) or a hypersensitive response (immediate threshold response) in prey [15]. Chemical (olfactory) cues given off by predators can alter prey behavior, activity, competition, and function within communities [3], [15]–[17]. Yet there are few examples of terrestrial insects avoiding predators via chemical cues [16]. For ant-herbivore interactions, it is very plausible that herbivores detect the volatile and profuse chemicals ants emit as pheromones to communicate alarm, territory marking, or trail making [10], [18]. The pheromone avoidance hypothesis suggests herbivores eavesdrop on ant communication pheromones to detect plants or leaves patrolled by ants and avoid them to avoid predation or confrontation [12]. Nevertheless empirical evidence for this hypothesis is scant and is only supported by *Oecophylla* ants in Asia and Africa [12]–[14]. For this hypothesis to be broadly supported and meaningful to arboreal insect communities world wide, examples of ant species that are broadly distributed geographically, taxonomically, and ecologically are needed.

Here, I tested if an herbivorous beetle (*Margaridisa* sp., Coleoptera: Chrysomelidae) avoids and inflicts less damage to leaves of its host tree, *Conostegia xalapensis* (Bonpl.) D. Don ex DC. (Melastomataceae), when leaves are marked with semiochemicals of three ant species; *Azteca instabilis* F. Smith (Dolichoderinae), *Camponotus textor* Forel (Formicinae), and *Solenopsis geminata* (Fabricius, 1804, Myrmicinae). For *A. instabilis*, I also tested if the length of time leaves were exposed to ant patrolling would influence the avoidance behavior and herbivory by the beetles. In other words, I tested whether beetles exhibited a threat sensitive response or a hypersensitive response to predator risk cues (ant semiochemicals) [15]. I predicted that the chemical cues of all ant species would alter the choice of beetles given that ants broadly limit herbivores on plants [5]–[7]. Additionally, I predicted that increasing the time of exposure to ant patrolling would result in an increase in avoidance of treatment leaves.

## Materials and Methods

I conducted all research at Finca Irlanda (15°11′N, 92°20′W; 900 m asl; 4500 mm/y rain), a 300-hectare shaded coffee plantation in the Soconusco region of Chiapas, Mexico in May - July of 2010 [19]. In the plantation and surrounding areas *C. xalapensis* is a common mid-sized tree (3–12 m). A flea beetle (*Margaridisa* sp.) consumes *C. xalapensis* and makes characteristic damage marks from the underside of leaves [20]. *Azteca instabilis* forms carton nests on the trunks and in crevices of *C. xalapensis* trees. Previously, it was reported that the presence of *A. instabilis* correlated with reduced beetle abundance and damage on this tree [20]. *Camponotus textor*, the Neotropical weaver ant, forms a silk nest by weaving host tree leaves together and is found in many tree species including *C. xalapensis* [21]. *Solenopsis geminata* is a ground-nesting species that is unlikely to interact with *C. xalapensis* or the herbivorous beetle due to its predominantly ground foraging habits. However because it both forages occasionally on vegetation and is an aggressive ant I included it in this study.

### Exp. 1 – Crushed ants (*A. instabilis*)

To determine if *A. instabilis* semiochemicals affect beetle choice, I performed a number of paired choice experiments. To control for any effects of host-plant quality, I used leaf pairs that were approximately equal in size, pre-existing damage, age, and located in the same compound pair on a branch. Additionally, I only collected leaves from trees with no observable ant activity. I randomly assigned one leaf to control and one to ant-treatment from the pair, and estimated leaf area (elliptical area = length÷2×width÷2×π) [22] and quantified pre-existing damage (number of feeding marks at the time the leaf was obtained). This first experiment was intended to determine if any ant chemicals impact beetle choice and reduce herbivory when deposited on leaves. I crushed 50 *A. instabilis* workers in a vial with a stir rod and applied the resulting liquid to one leaf (ant-treatment) by dotting 10 spots of the liquid on the underside of one *C. xalapensis* leaf with a cotton swab. I dotted the control leaf with a water-moistened cotton swab (*N* = 11 pairs). Next I placed both leaves within a cylindrical plastic container (8 cm height, 11 cm diameter) with 10 beetles and recorded the number of beetles in feeding position (undersides of leaf) three times over 24 h. Beetles were not starved. After 24 h, I calculated the number of new feeding marks and mean (across the three time points) number of beetles per leaf. Leaf size has the potential to influence the outcome of choice tests because beetles are more likely to occupy leaves of greater area. For example, across all samples of all experiments conducted in this study the mean number of beetles per leaf (*R* = 0.389, *N* = 506, *P*<0.001) and mean number of damage per leaf (*R* = 0.386, *N* = 498, *P*<0.001) correlated positively with leaf area (cm^2^). Therefore I converted both the number of beetles and the number of damage per leaf to values per cm^2^ leaf area. To compare beetle choice between treatments, I performed paired t-tests for three dependent variables: pre-existing damage (per cm^2^), beetles (per cm^2^), and damage (per cm^2^) (Table 1) using SPSS 16.0 [23]. I tested for normality and transformed the variables pre-existing damage, beetles, and damage with a square root transformation to meet the assumptions of normality.

**Table 1:** Experiments performed to test the effects of ant semiochemicals and to test the effects of leaf damage on herbivore choice.

Experiment	Ant species	Experimental design	Analysis (full models)
Exp. 1 -Crushed ants	*A. instabilis*	Crushed ants vs. water control; *N* = 11 leaf pairs	Paired t-test: Beetles, damage, pre-existing damage
Exp. 2 -Previously patrolled	*A. instabilis*	Previously patrolled treatment vs. control; *N* = 30 leaf pairs, *N* = 3 nests	GLM*: Fixed- treatment, nest, treat×nest, pre-existing damage per cm^2^ (covariate)
Exp. 3 -Tree experiment	*A. instabilis*	Leaf of *A. instabilis* nest tree vs. leaf of non-*A. instabilis* tree; *N* = 40 leaf pairs, *N* = 11 tree pairs	GLMM*: Random- leaf pair, tree pair; fixed- treatment, pre-existing damage per cm^2^ (covariate)
Exp. 4 -Previously patrolled	*C. textor*	Previously patrolled treatment vs. control; *N* = 33 leaf pairs (beetles), *N* = 29 for leaf pairs (damage), *N* = 4 nests	GLMM*: Random- leaf pair; fixed- treatment, pre-existing damage per cm^2^ (covariate)
Exp. 5 -Previously patrolled	*S. geminata*	Previously patrolled treatment vs. control; *N* = 30 leaf pairs, *N* = 2 nests	GLMM*: Random- leaf pair; fixed- treatment, pre-existing damage per cm^2^ (covariate)
Exp. 6 -Time exposed experiment	*A. instabilis*	Previously patrolled treatment vs. control and 0-, 5-, 30-, 90-, 180-min. exposure-length treatment; *N* = 101 leaf pairs	GLM*: Fixed- treatment, exposure-length, Treat×exposure-length, pre-existing damage per cm^2^ (covariate)
Exp. 7 -Manual damage	None	Manually damaged leaf vs. control; *N* = 17	Paired t-test: Beetles, damage, pre-existing damage
Exp. 8 -Pre-existing damage	None	Leaf with high vs. leaf with low pre-existing damage; *N* = 21	Paired t-test: Beetles, damage, pre-existing damage

*For each experiment, analysis was conducted with the dependent variables beetles (per cm^2^) and damage (per cm^2^). Factors listed were included in full models; some statistically non-significant factors were removed to improve the goodness of fit.

### Exp. 2 – Previously patrolled (*A. instabilis*)

In a second experiment, I aimed to determine if ant semiochemicals given off freely by ants would alter herbivore choice by only exposing ant-treatment leaves to ant patrolling. I placed ant-treatment leaves (undersides facing up) in a large plastic box (approximately 0.5 m×0.25 m×0.25 m) containing an active nest of *A. instabilis* (3 independent nests used). Ants continually patrolled treatment leaves at a rate of approximately 5–50 ants crossing each leaf per minute within a nest box. Leaves were left exposed to ant patrolling for 2.5–3 h exposure time. Meanwhile, I placed control leaves in a separate plastic box nearby without ants (*N* = 30 pairs). After the exposure time, I brought the two leaves of each pair back together and conducted beetle choice tests as described in Exp. 1. To determine the effect of the previously patrolled treatment, I compared mean beetles (per cm^2^) and damage (per cm^2^) with general linear mixed models (GLMM) in SPSS 16.0 [23]. GLMMs allowed me to maintain pair-wise design of the experimental setup, but also include other fixed factors and covariates. However, estimation of the covariance matrix *D* was not positive definite indicating that the validity of the results cannot be ascertained [24]. This may arise from the nature of data set being analyzed, the similarity between observations within a given level of a random factor, or during estimation of covariance parameters in the matrix when iterative estimation routines converge to a value that lies near to or outside the boundary of the parameter space [24]. I performed trouble shooting steps to modify the GLMMs by increasing the maximum number of iterations and the maximum number of step-halving within the Restricted Maximum Likelihood (REML) estimation algorithm and also by rescaling the covariate included in the model [24], however neither method changed the outcome. Therefore, I removed the random blocking factor “leaf pair” from the model and thereby converted the model to a General Linear Model (GLM) with no random effects [24]. Although removing the paired structure of the model reduced the power of the statistical test, the outcome of the GLM was similar to a paired t-test (without additional factors or a covariate), therefore I felt confident in results produced by the GLM. In the GLM, I included treatment and nest as fixed factors, the interaction between the two factors, and pre-existing damage (per cm^2^) as a covariate (Table 1). I then obtained a best-fit model by comparing Akaike Information Criterion (AIC) of reduced models. I reduced models by removal of each non-significant factor that lowered the goodness of fit. I tested for normality with the residuals from the models using a Kolmogorov-Smirnov Test, comparing q-q plots, and plotting residuals against predicted values [25].

### Exp. 3 – Tree experiment (*A. instabilis*)

To determine if the effects of ant semiochemicals alter herbivore choice on trees occupied by ants, I compared beetle choice between leaves from *C. xalapensis* trees with *A. instabilis* nests and leaves from the nearest trees without *A. instabilis* (*N* = 40 leaf pairs; *N* = 11 tree pairs). I first located the 11 pairs of trees with and without *A. instabilis* nests. No two trees were within 10 m of one another. The goal of this experiment was to determine if *A. instabilis* semiochemical marks impacted beetle choice in areas of high *A. instabilis* activity (near base of tree) only because the effect of *A. instabilis* on beetles in areas of low activity appears to be weaker [20]. Therefore, I first disturbed the *A. instabilis* nests by beating the trunk to determine the location of the nest, determine which leaves received high activity (<1 m from nest site), and to ensure recent activity on leaves. I also searched and beat the trunk of control trees to ensure that it was free of any observable ant activity. I paired one high-activity leaf from an *A. instabilis* tree to one leaf of similar size and age from a tree without *A. instabilis*. I could not control for pre-existing damage because damage on *C. xalapensis* trees without *A. instabilis* averages 20–25 times higher than on trees with *A. instabilis* [20]. I compared at least 3 leaf pairs for each ant - no ant tree pair (except for one case where only 1 leaf pair was compared). With each leaf pair I conducted beetle choice test as described in Exp. 1. To determine the influence of *A. instabilis* tree treatment, I compared mean beetles (per cm^2^) and damage (per cm^2^) with GLMMs (Table 1). I included leaf pair and tree pair in the model as random effect factors. *Azteca instabilis* leaf treatment was included as a fixed factor and pre-existing damage (per cm^2^) was a covariate in the model. I performed type III F-tests of significance for the main effects using REML to estimate the fixed effect parameters in addition to the variance of the random effects [24]. As in Exp. 2, I reduced full models to best-fit models using AIC. I tested for normality of the data as described in Exp. 2.

### Exp. 4 – previously patrolled (*C. textor*)

To determine if the effects of ant semiochemicals on herbivores is widespread across the ant family, I repeated methods as in the Exp. 2 described above using *C. textor*. For *C. textor*, I exposed leaves to *C. textor* patrolling for 2.5–3 h, by placing them in a plastic box with a *C. textor* silk nest (four independent nests used). I observed *C. textor* workers patrolling experimental leaves. Meanwhile control leaves were un-exposed in a separate box. After this period, I subjected leaf pairs to beetle choice tests as described in Exp. 1. To determine the influence of the previously patrolled treatment on mean beetles (per cm^2^) and damage (per cm^2^), I compared means with GLMMs as in Exp. 3 (*N* = 33 pairs for beetles and *N* = 29 pairs for damage comparisons). In each model I included a leaf pair as a random effect factor, treatment as a fixed factor, and pre-existing damage (per cm^2^) as a covariate in the models (Table 1). As in Exp. 2, I reduced full models to best-fit models using AIC and tested for normality.

### Exp. 5 – Previously patrolled (*S. geminata*)

To determine if the effects of ant semiochemicals on herbivores occurs in *S. geminata*, I repeated methods as in the Exp. 2 and 4 described above using *S. geminata* ants. A highly active portion of a *S. geminata* nest was shoveled into a plastic container lined with tanglefoot (The Tanglefoot Co., Grand Rapids, MI, USA) using a spade. I exposed leaves to *S. geminata* patrolling for 2.5–3 h, by placing them in a plastic box adjacent the *S. geminata* nest (two independent nests used). I observed ants patrolling experimental leaves. Meanwhile control leaves were un-exposed in a separate container. After this period, I subjected leaf pairs to beetle choice tests as described in Exp. 1 (*N* = 30 pairs for *S. geminata*). To determine the influence of the previously patrolled treatment (*S. geminata* patrolled versus control), I compared mean beetles (per cm^2^) and damage (per cm^2^) with GLMMs as in Exp. 3 (Table 1). In each model I included leaf pair as a random effect factor, ant-treatment and nest as a fixed factors, the interaction between these two main effects, and pre-existing damage (per cm^2^) was incorporated into the model as a covariate. As in Exp. 2, I reduced full models to best-fit models using AIC and tested for normality.

### Exp. 6 – Time-exposed experiment (*A. instabilis*)

This experiment aimed to determine how the level of ant semiochemicals deposited on the leaf affected beetle choice. To determine this relationship I varied the amount of time ant-treatment leaves were exposed to *A. instabilis* patrolling from 0- (*N* = 23), 5- (*N* = 22), 30- (*N* = 17), 90- (*N* = 21), and 180- (*N* = 18) minutes (exposure-length treatment) and compared the beetle choice between each treatment leaf and a respective control leaf (placed in a box without ants). I began the 180-min. exposure treatment first, and sequentially added the 90-, 30-, and 5- min. treatments to the box with *A. instabilis* ants. This ensured that all exposure-length treatments finished simultaneously and that all choice tests started at the same time. After exposure durations were complete, I brought control and ant-treatment leaf pairs together and initiated beetle choice experiments as described in Exp. 1. To determine the effect of altering the exposure-length of ant-treatments on mean beetles (per cm^2^) and damage (per cm^2^), I compared means across treatments with GLMMs, however as in Exp. 2, the Hessian matrix of these models was not positive definite. I therefore followed problem shooting steps outline in Exp. 2. In final, I removed the random effect “leaf pair” from the models and converted the models to GLMs. In the GLMs, I incorporated ant-treatment (ant-exposed versus control) as a fixed effect, exposure-length (0-, 5-, 30-, 90-, 180-min.) as fixed effect, the interaction between main effects, and pre-existing damage (per cm^2^) as a covariate (Table 1). As in Exp. 2, I reduced full models to best-fit models using AIC. To determine the effect of ant treatment at each of the exposure-length treatments I used pair-wise comparisons of the estimated marginal means. The pair-wise comparisons were t-tests with a Bonferroni correction for multiple comparisons. I also compared the effect of exposure-length treatments across both ant and control treatments with pair-wise comparisons in the same manner. I tested for normality as described in Exp. 2 and square root transformed both beetles (per cm^2^) and damage (per cm^2^) dependent variables.

### Exp. 7 - Manual damage

The level of pre-existing damage on leaves confounded the results of a couple experiments. To determine the effect of pre-existing damage on beetle choice, I determined the response of *Margaridisa* sp. flea beetles to manually damaged leaves relative to undamaged leaves (manual damage experiment). I collected leaves controlling for the same factors described in Exp. 1. I inflicted damage to one leaf by piercing it 20 times with a needle. The control leaves were left undamaged. Then I conducted beetle choice tests as described in Exp. 1. I compared mean differences between control and treatment with a paired t-test for three dependent variables: pre-existing damage (per cm^2^), beetles (per cm^2^), and damage (per cm^2^) (*N* = 17 pairs, Table 1). I tested for normality and square root transformed the dependent variable pre-existing damage (per cm^2^) to meet the assumptions of normality.

### Exp. 8 – Pre-existing damage

To further investigate the effect of pre-existing damage on beetle choice, I compared choice between leaves of high and low pre-existing damage (*N* = 21 pairs) (pre-existing damage experiment). Leaves were collected as in Exp. 1, however in this experiment I looked for leaf pairs with large differences in pre-existing damage. Choice tests were performed as in Exp. 1. I compared mean differences between high and low pre-existing damage treatment with a paired t-test for three dependent variables: pre-existing damage (per cm^2^), beetles (per cm^2^), and damage (per cm^2^) (Table 1). I tested for normality and found the need to transform the dependent variables pre-existing damage (per cm^2^), beetles (per cm^2^), and damage (per cm^2^) with the square root transformation to meet the assumptions of normality. All statistical tests in all experiments were conducted with SPSS (16.0).

## Results

### Exp. 1 – Crushed ants (*A. instabilis*)

**Figure 1:**
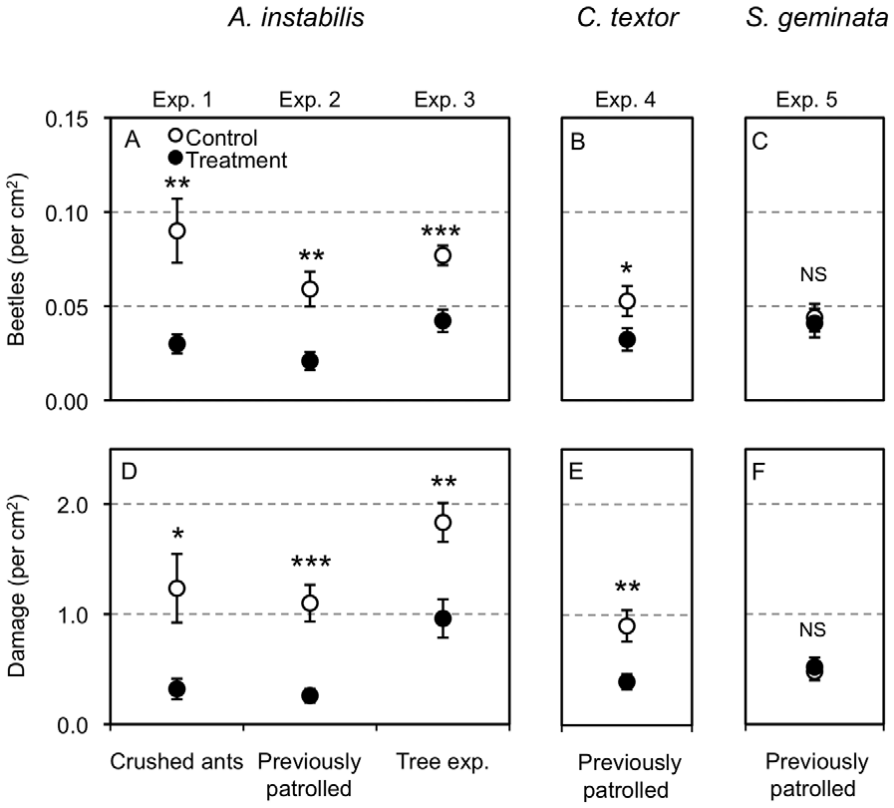
The effect of ant semiochemical treatments on beetle choice and herbivory. Experiments 1–5 treatment means (± SE) for the number of beetles per cm^2^ (A–C) and mean damage per cm^2^ (D–F). Specifically, Exp. 1 – crushed *A. instabilis* ant treatment versus control experiment (A,D), Exp. 2 -leaves previously patrolled by *A. instabilis* versus control experiment (A,D), Exp. 3 -leaves from trees with *A. instabilis* nests versus leaves from trees without nests (A,D), Exp. 4 -leaves previously patrolled by *C. textor* versus control leaves experiment (B,E), and Exp. 5 -leaves previously patrolled by *S. geminata* versus control leaves experiment (C,F). The x-axis represents the method of ant-treatment for each experiment. Statistical differences between ant and control treatment means are represented as: NS = *P*>0.05, * = *P*<0.05, ** = *P*<0.01, *** = *P*<0.001.

The crushed ants experiment revealed that ant chemicals influence herbivore choice and herbivory. Water swabbed control leaves had 3 times more beetles (*t* = 3.2, *df* = 10, *P* = 0.01) and 3.8 times more damage (*t* = 2.6, *df* = 10, *P* = 0.025) than the crushed ant-treated leaves (Fig. 1A,D). Although ant-treatment leaves had higher pre-existing damage (*t* = −2.3, *df* = 10, *P* = 0.047) than control leaves, pre-existing damage was not correlated with the number of beetles (*R* = −0.024, *df* = 21, *P* = 0.916) or the amount of damage (*R* = −0.067, *df* = 21, *P* = 0.769).

### Exp. 2 – Previously patrolled (*A. instabilis*)

Allowing ants to patrol treatment leaves for 3 h prior to choice tests significantly reduced herbivore choice and herbivory. Control leaves had 2.9 times more beetles and 2.1 times more damage than leaves previously patrolled by *A. instabilis* (Table 2, Fig. 1A,D). There was no effect of the nest used, no interaction between nest and ant-treatment factors (statistics reported in Table S1). There was no significant effect of including pre-existing damage as a covariate in the beetle model (Table S1). But pre-existing damage was a significant factor in the beetle model and it increased with damage (Table 2). Comparison of AIC of reduced models revealed the best-fit beetle model included only ant-treatment, whereas the best-fit damage model included ant-treatment and the covariate pre-existing damage (Table 2).

**Table 2:** The effect of ant semiochemical treatments on beetle choice and herbivory (best-fit models Exp. 2–5)†.

	Beetles (per cm^2^)			Damage (per cm^2^)		
Experiment	*df* *	*F*	*P*	*df* *	*F*	*P*
***Azteca instabilis***						
**Exp. 2 Previously patrolled (GLM)**						
Intercept	1,58	58.8	<0.001	1,57	12.2	0.001
Treatment	1,58	13.4	0.001	1,57	21.3	<0.001
Pre-existing damage (per cm^2^)	-	-	-	1,57	4.0	0.050
**Exp. 3 Tree experiment (GLMM)**						
Intercept	1,39	168.9	<0.001	1,39	98.4	<0.001
Treatment	1,39	29.5	<0.001	1,39	17.1	0.001
***Camponotus textor***						
**Exp. 4 Previously patrolled (GLMM)**						
Intercept	1,32	71.6	<0.001	1,35	53.6	<0.001
Treatment	1,32	4.3	0.047	1,25	8.8	0.007
Nest	-	-	-	3,25	0.3	0.805
Treatment×Nest	-	-	-	3,25	1.4	0.353
***Solenopsis geminata***						
**Exp. 5 Previously patrolled (GLMM)**						
Intercept	1,27	52.2	<0.001	1,28	60.6	<0.001
Treatment	1,29	0.1	0.751	1,31	0.1	0.709

*For GLMM *df* = numerator,denominator; For GLM *df* = among group *df*, error *df*.

† Dashes (-) replace statistics of factors not included in the best-fit model.

### Exp. 3 – Tree experiment (*A. instabilis*)

Leaves from trees with active *A. instabilis* nests were less preferred than leaves from trees without *A. instabilis* nests. Leaves from trees without *A. instabilis* had 1.8 times more beetles and 2.2 times more damage than leaves from trees with *A. instabilis* after beetle choice tests (Table 2, Fig. 1A,D). Although trees without *A. instabilis* (139±21, mean ± SE) nests had more pre-existing damage than trees with nests (32±5) (Paired *t* = 4.8, *P* = 0.001), the covariate pre-existing damage (per cm^2^) was not a significant factor in the models (Table S1). Comparison of AIC of reduced models revealed the best-fit beetle and damage model included only ant-treatment (Table 2).

### Exp. 4 – previously patrolled (*C. textor*)

The *C. textor* semiochemical treatment also reduced beetle choice and herbivory. Control leaves had 1.6 times more beetles and 2.2 times more damage than leaves previously patrolled by *C. textor* (Table 2, Fig. 1B,E). Nest, the interaction between nest and ant-treatment, and pre-existing damage were not significant factors in the beetle and damage models (Table S1). Comparison of AIC of reduced models revealed the best-fit beetle model only included ant-treatment, whereas the best-fit damage model included ant-treatment, nest, and their interaction (Table 2).

### Exp. 5 – Previously patrolled (*S. geminata*)

Although *A. instabilis* and *C. textor* semiochemical treatment appeared to alter herbivore choice, *S. geminata* semiochemical treatment had no effect. Control leaves had 7% more beetles and 8% less damage relative to leaves previously patrolled by *S. geminata*; these differences were not statistically significant (Table 2, Fig. 1C,F). Further, there was no significant effect of including nest, nest by ant-treatment interaction, and pre-existing damage (covariate) in the beetle or damage models (Table S1). Comparison of AIC of reduced models revealed the best-fit beetle and damage models only included ant-treatment factors (Table 2).

### Exp. 6 – Time-exposed experiment (*A. instabilis*)

**Figure 2:**
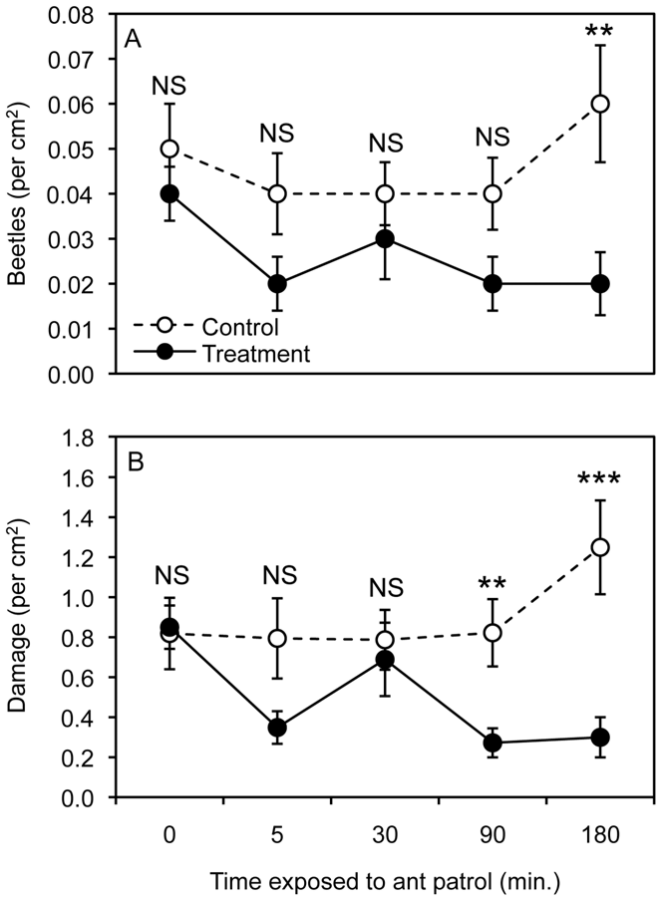
The effect of ant-treatment exposure-length on beetle choice and herbivory. Experiment 6 treatment means (± SE) for the number of beetles per cm^2^ (A) and damage marks per cm^2^ (B) on control leaves and leaves exposed to *A. instabilis* patrolling for 0-, 5-, 30-, 90-, and 180-min. prior to choice test. Statistical differences between ant and control treatment means are represented as: NS = *P*>0.05, * = *P*<0.05, ** = *P*<0.01, *** = *P*<0.001.

The time-exposed experiment revealed complex effects of the exposure-length treatment on beetle choice and herbivory. For the number of beetles, there was a significant effect of ant-treatment, but no effect of the exposure-length (0-, 5-, 30-, 90-, 180-min.) of ant-treatments and no interaction between main effects (Table 3, Fig. 2A). Pre-existing damage was not a significant covariate in the model. Comparison of AIC of reduced models revealed the best-fit beetle model only included ant-treatment, exposure-length treatment, and their interaction (Table S2). As found in the above experiments, exposure to previous *A. instabilis* patrolling (ant-treatment versus control) reduced the number of beetles (per cm^2^) relative to control treatment across all exposure durations combine. Pair-wise comparisons (t-test) of estimated marginal means (Bonferroni corrected) revealed control leaves had more beetles (per cm^2^) than ant-treatment leaves for the 180-min. treatment (*P* = 0.003), however there was no difference between control and ant-treatment leaves for the 0-min. (*P* = 0.894), 5-min. (*P* = 0.069), 30-min. (*P* = 0.234), and 90-min. (*P* = 0.165) exposure-length treatments.

**Table 3:** The effect of ant treatment exposure-length on beetle choice and herbivory (best-fit models Exp. 6)†.

	Beetles (per cm^2^)			Damage (per cm^2^)		
GLM	*df* *	*F*	*P*	*df* *	*F*	*P*
Intercept	1,192	192.5	<0.001	1,191	246.9	<0.001
Treatment	1,192	10.6	0.001	1,191	18.5	<0.001
Exposure-length	4,192	1.3	0.296	4,191	2.7	0.031
Treatment×Exposure-length	4,192	1.4	0.232	4,191	4.5	0.002
Pre-existing damage (per cm^2^)	-	-	-	1,191	4.8	0.029

*df = among group *df*, error *df*.

† Dashes (-) replace statistics of factors not included in the best-fit model.

For the amount of damage across treatment leaves, the GLM revealed that there was a significant effect of ant-treatment, a significant effect of exposure-length treatment, a significant interaction between main effects, and pre-existing damage was a significant covariate in the model that increased with increasing damage (Table 3, Fig. 2B). Specifically, pair-wise comparisons (t-test) of estimated marginal means (Bonferroni corrected) revealed control leaves had more damage than ant-treatment leaves in the 180-min. (*P*<0.001) and 90-min. (*P* = 0.001) exposure-length treatments, but there was no difference between control and ant-treatment for the 0-min. (*P* = 0.418), 5-min. (*P* = 0.083), and 30-min. (*P* = 0.507) exposure-length treatments. The interaction between main effects was driven by differences in damage to leaves in exposure-length treatments of the ant-treatment group, but not the control-treatment group. Within the ant-treatment group, there was less damage in the 5-min. (*P* = 0.012), 90-min. (*P* = 0.001), and 180-min. (*P* = 0.001) treatments relative to the 0-min. exposure-length treatment. All other pair-wise comparisons between exposure-length treatments within the ant-treatment group were not statistically significant. There were no differences between exposure-length treatments in the control treatment group (example: mean damage did not differ between control 0-min. exposure-length treatment and control 180-min. exposure-length treatment; *P* = 1.0).

### Exp. 7 - Manual damage

**Figure 3:**
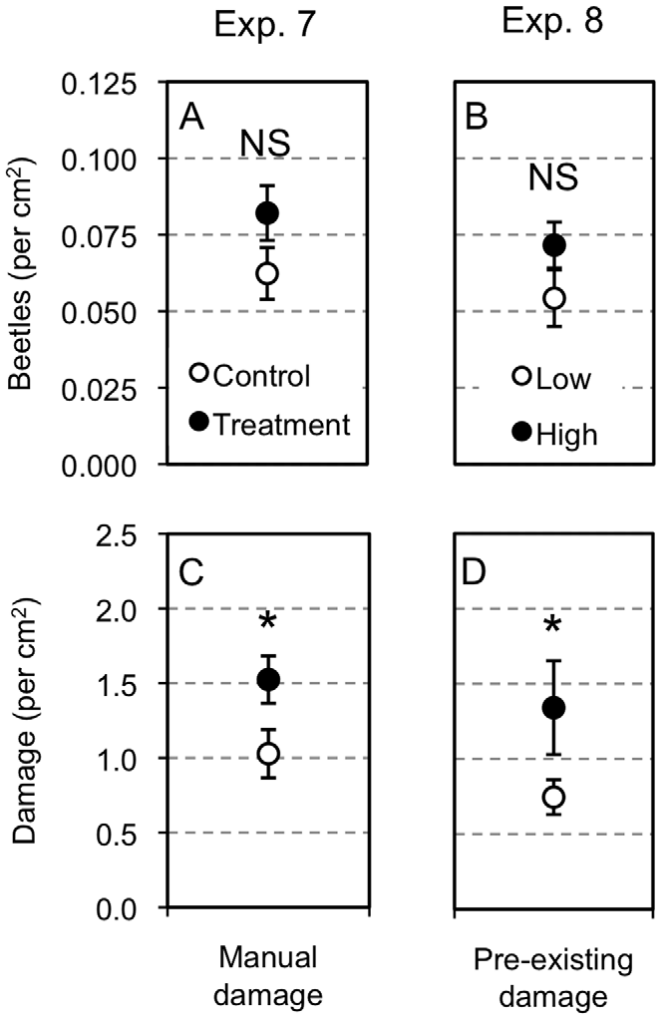
Effect of leaf damage on beetle choice and herbivory. Experiments 7–8 treatment means (± SE) number of beetles per cm^2^ (A,B) and damage per cm^2^ (C,D). Exp. 7 compares manually damaged leaves versus un-damaged control leaves (A,C). Exp. 8 compares leaves with high- versus leaves with low-pre-existing damage (B,D). Statistical differences between ant and control treatment means are represented as: NS = *P*>0.05, * = *P*<0.05, ** = *P*<0.01, *** = *P*<0.001.

Manually damaging leaves had weak effects on beetle choice and herbivory (Fig. 3A,C). Although there was 31% more beetles on manually damaged leaves compared to control leaves this difference was not significant (*t* = −1.5, *df* = 16, *P* = 0.164, Fig. 3A). However, the amount of beetle-inflicted damage on manually damaged leaves was 48% higher than on control leaves and this difference was statistically significant (*t* = −2.4, *df* = 16, *P* = 0.032, Fig. 3C). There was no difference in the amount of pre-existing damage between treatments (*t* = 0.06, *df* = 16, *P* = 0.951).

### Exp. 8 – Pre-existing damage

Comparing the leaves with high and low pre-existing damage also revealed weak influences of leaf damage on beetle choice and herbivory (Fig. 3B,D). Although there were 32% more beetles on high-treatment leaves relative to low-treatment leaves, this difference was only marginal (*t* = 2.0, *df* = 20, *P* = 0.056, Fig. 3B). The amount of damage inflicted by beetle was 80% greater on high-treatment relative to low-treatment leaves (*t* = 2.1, *df* = 20, *P* = 0.049, Fig. 3D). Mean pre-existing damage was 17 times higher on the high-treatment leaves relative to low treatment leaves confirming the categories of the pre-existing damage treatments (*t* = 7.6, *df* = 20, *P*<0.001).

## Discussion

For both *A. instabilis* and *C. textor*, my results support the hypothesis that ant semiochemical markings alter herbivore choice. Chemical compounds from crushed *A. instabilis* individuals (Exp. 1), chemicals given off freely by *A. instabilis* and *C. textor* (Exp. 2, 4), and leaves collected from trees with active *A. instabilis* nests all had fewer beetles and less damage than control leaves without chemicals or leaves from trees without ants (Exp. 3). In contrast to the above results, *S. geminata* treatment leaves did not differ from control leaves (Fig. 1C,D), which could imply that the herbivorous beetle did not recognize *S. geminata* semiochemicals as potential threats. However, there are many possible explanations for this result. For instance, it could be that the herbivorous beetle cannot detect *S. geminata*'s semiochemical or *S. geminata* did not mark leaves with semiochemicals. There also could be experimental errors. For example, if control leaves did differ for damage or beetle number across ant experiments (*A. instabilis*, *C. textor*, and *S. geminata*) and treatment leaves did not differ across ant species experiments then this would suggest that some other factor is responsible for the lack of ant effect in the *S. geminata* experiment. Indeed, control leaves in the *S. geminata* experiment (Exp. 5) had 2.2 times less damage than control leaves of the *A. instabilis* experiment (Tukey HSD, *P* = 0.004, Exp. 2) (*F_2,86_* = 5.5, *P* = 0.005). *Solenopsis geminata* control leaves also had 87% less damage than control leaves in the *C. textor* experiment (Exp. 4), although this difference was not statistically significant (*P* = 0.079, Exp. 4). Despite these differences in controls, there were also differences in damage across ant treatment leaves. There was less damage to the treatment leaves of *A. instabilis* experiment than to the treatment leaves of the *S. geminata* experiment (*P* = 0.035). Yet there was no differences between *C. textor* and *S. geminata* treatment leaves (*P* = 0.38) (*F_2,86_* = 3.2, *P* = 0.046). Controls did not differ in the number of beetles across ant species experiments (*F_2,90_* = 0.8, *P* = 0.442). Although not quite straightforward, these findings reduce the validity of the controls in the *S. geminata* experiment and suggest they could be biasing the results. Therefore care should be taken when drawing the conclusion that *S. geminata* does not have chemical effects on this herbivore. Further experimentation may help elucidate the existence or absence of chemical effects of *S. geminata* on this herbivore.

Predator avoidance behavior by prey can form a trade-off between the benefits of successful predator detection and the costs associated with other fitness-related activities [15]. Prey species often exhibit variation in their response to predator risk cues, either exhibiting hypersensitivity or threat sensitivity to predator cues [15]. The results of Exp. 6 (time-exposed Exp.) show that previous ant activity on leaves must have occurred for at least 90-min. to observe effects of ant semiochemicals on herbivore feeding (Fig. 2). This implies that beetle response to ant predator cues is not immediate and therefore follows a pattern more similar to threat sensitivity relative to a hypersensitive response. In other words, increased ant semiochemical concentration on a leaf should result in an increased response of the herbivorous beetle. If beetles exhibited a hypersensitive response, the 5-min. treatment should have had significant effects and of similar magnitude as the 180-min. treatment. It should be noted that beetle response would be much clearer, if not for the strange deviation from pattern at the 30-min. exposure-length treatment (Fig. 2). This deviation is unexplained, but may be due, by chance, to differences in beetle behavior or leaf quality within this treatment. It also could be due to experimental error of some type. I can think of no biological mechanism to explain this pattern. In a similar study, with *Oecophylla* ants, Van Mele et al. [14] found a negative correlation between fruit fly damage (in laboratory choice tests) on mangos collected at different distances from *Oecophylla longinoda* ant nests in mango trees (an indirect measurement of ant semiochemical concentration). All ants were removed from mangos before choice tests therefore the response of fruit flies to distance treatments was likely due to the effect of decreased ant deposition of semiochemicals with increased distance away from the nest [12], [14]. These results suggest that fruit flies exhibit a threat sensitive response pattern as well. However, it should be noted that neither the current study nor Van Mele et al. [14] actually measured concentrations of ant semiochemicals on leaves therefore conclusions from these data are not certain. More tests are needed to truly understand how variation in actual concentrations of ant semiochemicals can drive changes in prey response.

This study also revealed the importance of other leaf traits for beetle choice on this host tree. Exp. 7 and 8 showed that manually damaging leaves and high densities of pre-existing damage on leaves increased beetle preference relative to control or low pre-existing damage, respectively. This attractive effect of leaf damage has been observed before for flea beetles and some other herbivores and is thought to a mate-finding mechanism for some species [26]–[27]. Additionally, leaf area (cm^2^) positively correlated with beetles and damage per leaf (see methods Exp. 1) across all studies combined and therefore likely is important to herbivore choice. It may be that larger leaves have a higher probability of beetles encountering them or that larger leaves produce more volatile compounds and therefore are more attractive. These factors could be important to the results of the ant semiochemical experiments considering in Exp. 6 pre-existing damage co-varied with the amount of damage inflicted by the beetles in the choice experiments. However, given that this study tested the hypothesis that ant semiochemicals deter herbivores across multiple experiments, I feel the results are robust and are not explained by differences in leaf traits.

To date only the semiochemicals of two ants species (of the same genera) are known to alter herbivore choice. The semiochemicals of *Oecophylla smaragdina* deterred chrysomelidae beetles and reduced damage to leaves [12] and the semiochemicals of *O. longinoda* similarly reduced the oviposition of fruit flies and damage to mangos [13]–[14]. Although *Oecophylla* ants are ecologically significant and widely distributed in Asia and Africa, they are a small group taxonomically. *Azteca* and *Camponotus* provide two new examples from diverse and ecologically important genera, suggesting that chemically driven trait-mediated effects of ants on herbivores may be widespread within the ant family. The *Camponotus* genus is one of the largest genera of ants within the entire family [10], [28] and many species foraging on trees have important ecological impacts on arboreal herbivore communities [7], [29]–[30]. *Azteca* ants are a ubiquitous component of most Neotropical forest communities [31] and are known for their ecological impacts on herbivores and indirect positive effects on plants [19]–[20], [31]–[34]. If herbivores avoid ants through semiochemical markings alone, there is great potential for further study of other ant-plant associations. Perhaps differences in the chemical composition of a given ant species may alter how herbivores respond to their chemical cues. Additionally, herbivore responses to ant cues may be a learned response and may depend on the frequency of interactions between herbivore and ant. Therefore ant traits like nest location (ground or arboreal) may be of great importance. Finally, perhaps the level of coevolution between ant, plant, and herbivore interactions may also contribute to variation in the strength of the trait-mediated interactions.

This study did not aim to determine which specific chemicals are responsible for the deterrent effects of ant semiochemicals. Ants produce a diversity of semiochemicals used in communication making it difficult to speculate which chemicals are important [10], [18], [35]. For *Oecophylla* ants, Offenberg et al. [12] suggested long-lasting territorial anal spot markings are likely the chemicals responsible for deterrent effects, although Van Mele et al. [14] argue trail pheromones from the Pavan's gland could also provide important chemical cues. For *A. instabilis* possible compounds include the strong smelling cyclopentanes and iridoids produced in the pygidial gland that act as alarm-defense pheromones [36]–[37] and are used as cues for *A. instabilis* phorid parasitoid flies [38]. Additionally, there a number of trail pheromones from the Pavan's gland that are poorly described [18] but longer lasting than pygidial compounds [39]. Given that volatile alarm pheromones dissipate rapidly into the environment, it is likely that the chemicals responsible for effects are long lasting trail pheromones because treatment effects maintained significance for at least 24 h. Less is known about the semiochemicals that *C. textor* produces, although some *Camponotus* species are well studied. Some *Camponotus* spp. emit large amounts of formic acid as a recruitment pheromone and as a defensive compound, which could be easily detected by herbivores [40]–[41]. Yet Dufour's and poison gland excretions include a wide diversity of possible chemical cues from undecane and other hydrocarbons, alcohols, ketones, and acetates [18], [41]. *Solenopsis geminata* also produces a diversity of chemical pheromones that are known to be important in other interactions with ants and ant parasitoids (phorid flies) [42]–[44]. *Solenopsis* spp. invest in alkaloid and proteinaceous toxins that they use to sting or to disseminate around themselves in defense [44]. Additionally, they produce a number trail pheromones from different glands used in recruitment to food sources [18], [42]. Description of the compounds responsible for chemical driven trait-mediated effects may aid in predictions of which ants are likely to have strong olfactory effects and which will not.

Strong olfactory driven trait-mediated effects appear to be rare in terrestrial arthropod interactions ([16]; but see [45]–[46]), although they are pervasive in aquatic systems [3], [16]–[17] and frequently observed in vertebrate terrestrial interactions [16], [47]–[49]. Here, I suggest for ants, the most abundant animals in the tropics, these interactions may be common and greatly important. First, many herbivores have well developed olfactory senses used to locate mates or host plants [26]–[27]. Second, ant chemical markings on plants used for communication are not subtle and are available for other organisms, such as herbivores, to detect and interpret [10], [12]–[14]. Third, there is a vast number of studies demonstrating that other organisms take advantage of ant semiochemicals in their interspecific interactions with ants. For example, ant-ant interactions are mediated by ant semiochemicals [50], hemipteran mutualists alter their behavior in the presence of chemicals of their ant-partners [51], and ant semiochemicals are important cues for ant parasites to locate their ant hosts [38], [43]. Therefore it should be no surprise that herbivores too have evolved to detect and avoid ant semiochemicals given the overwhelming evidence that ants prey upon or attack herbivores on plants [2], [4]–[7]. This study provides two new examples of ant chemical cues altering herbivore choice and suggests these effects may be common within ant-herbivore interactions. Future studies should aim to determine how differences in chemical composition of ant species results in differences in their chemical effect on herbivores. Finally, understanding how different herbivore species respond differently to ant chemicals will also be vitally important.

## Supporting Information

Table S1 **The effect of ant semiochemical treatments on beetle choice and herbivory (full models Exp. 2–5).**
(DOC)

Table S2 **The effect of ant-treatment exposure-length on beetle choice and herbivory (full models Exp. 6).**
(DOC)
