# Supplementary material for: Do Herbivores Eavesdrop on Ant Chemical Communication to Avoid Predation?
Source: PLoS One. 2012 Jan 3;7(1):e28703. doi: 10.1371/journal.pone.0028703 (PMC3250387; doi:10.1371/journal.pone.0028703)
Supplement: Table S1 — The effect of ant semiochemical treatments on beetle choice and herbivory (full models Exp. 2–5). (DOC) [file pone.0028703.s001.doc]

**Table S1 The effect of ant semiochemical treatments on beetle choice and herbivory (full models Exp. 2-5).**

|  | Beetles (per cm^2^) | | | Damage (per cm^2^) | | |
| --- | --- | --- | --- | --- | --- | --- |
| **Experiment** | *df** | *F* | *P* | *df** | *F* | *P* |
| ***Azteca instabilis*** |  |  |  |  |  |  |
| **Exp. 2 Previously patrolled (GLM)** |  |  |  |  |  |  |
| Intercept | 1,53 | 19.5 | <0.001 | 1,53 | 16 | <0.001 |
| Treatment | 1,53 | 12.4 | 0.001 | 1,53 | 21 | <0.001 |
| Nest | 2,53 | 0.4 | 0.661 | 2,53 | 1 | 0.393 |
| Treatment × Nest | 2,53 | 0.1 | 0.876 | 2,53 | 0.3 | 0.727 |
| Pre-existing damage (per cm^2^) | 1,53 | 0.1 | 0.732 | 1,53 | 0.9 | 0.346 |
| **Exp. 3 Tree experiment (GLMM)** |  |  |  |  |  |  |
| Intercept | 1,74 | 89.2 | <0.001 | 1,74 | 52.2 | <0.001 |
| Treatment | 1,48 | 19 | <0.001 | 1,50 | 12.6 | 0.001 |
| Pre-existing damage (per cm^2^) | 1,63 | 0.5 | 0.491 | 1,66 | 0.6 | 0.429 |
| ***Camponotus textor*** |  |  |  |  |  |  |
| **Exp. 4 Previously patrolled (GLMM)** |  |  |  |  |  |  |
| Intercept | 1,37 | 44.5 | <0.001 | 1,31 | 41.5 | <0.001 |
| Treatment | 1,29 | 0.8 | 0.386 | 1,27 | 7 | 0.014 |
| Nest | 3,29 | 0.8 | 0.51 | 3,26 | 0.3 | 0.805 |
| Treatment × Nest | 3,29 | 1.3 | 0.305 | 3,26 | 1.4 | 0.261 |
| Pre-existing damage (per cm^2^) | 1,56 | 2.4 | 0.126 | 1,44 | 1.3 | 0.261 |
| *Solenopsis geminata* |  |  |  |  |  |  |
| **Exp. 5 Previously patrolled (GLMM)** |  |  |  |  |  |  |
| Intercept | 1,31 | 32.6 | <0.001 | 1,31 | 36.2 | <0.001 |
| Treatment | 1,29 | 0.1 | 0.778 | 1,29 | 0.1 | 0.743 |
| Nest | 1,28 | 0.2 | 0.697 | 1,28 | 1 | 0.319 |
| Treatment × Nest | 1,28 | 0.007 | 0.935 | 1,28 | 0.2 | 0.654 |
| Pre-existing damage (per cm^2^) | 1,43 | 0.01 | 0.922 | 1,41 | 0.8 | 0.39 |

*For GLMM *df* = numerator,denominator; For GLM *df* = among group *df*, error *df*
