# Supplementary material for: Do Herbivores Eavesdrop on Ant Chemical Communication to Avoid Predation?
Source: PLoS One. 2012 Jan 3;7(1):e28703. doi: 10.1371/journal.pone.0028703 (PMC3250387; doi:10.1371/journal.pone.0028703)
Supplement: Table S2 — The effect of ant-treatment exposure-length on beetle choice and herbivory (full models Exp. 6). (DOC) [file pone.0028703.s002.doc]

**Table S2 The effect of ant-treatment exposure-length on beetle choice and herbivory (full models Exp. 6).**

|  | Beetles (per cm^2^) | | | Damage (per cm^2^) | | |
| --- | --- | --- | --- | --- | --- | --- |
| GLM | *df** | *F* | *P* | *df** | *F* | *P* |
| Intercept | 1,191 | 211.1 | <0.001 | 1,191 | 246.9 | <0.001 |
| Treatment | 1,191 | 11.9 | 0.001 | 1,191 | 18.5 | <0.001 |
| Exposure-length | 4,191 | 1.6 | 0.183 | 4,191 | 2.7 | 0.031 |
| Treatment × Exposure-length | 4,191 | 1.2 | 0.320 | 4,191 | 4.5 | 0.002 |
| Pre-existing damage (per cm^2^) | 1,191 | 1.5 | 0.226 | 1,191 | 4.8 | 0.029 |

**df* = among group *df*, error *df*
